# Exosomal miR-500a-5p derived from cancer-associated fibroblasts promotes breast cancer cell proliferation and metastasis through targeting USP28

**DOI:** 10.7150/thno.53412

**Published:** 2021-02-06

**Authors:** Bing Chen, Yuting Sang, Xiaojin Song, Dong Zhang, Lijuan Wang, Wenjing Zhao, Yiran Liang, Ning Zhang, Qifeng Yang

**Affiliations:** 1 Department of Breast Surgery, General Surgery, Qilu Hospital of Shandong University.; 2 Pathology Tissue Bank, Qilu Hospital of Shandong University.; 3 Research Institute of Breast Cancer, Shandong University.

**Keywords:** cancer-associated fibroblasts, exosome, miR-500a-5p, breast cancer, USP28

## Abstract

The tumor microenvironment contributes to tumor progression and metastasis. Cancer-associated fibroblasts (CAFs) form a major cellular component of the tumor microenvironment. In this study, we further explored the mechanisms underlying the tumor-promoting roles of CAFs.

**Methods:** Patient-derived CAFs and normal fibroblasts (NFs) were isolated from breast carcinomas and adjacent normal breast tissue. Exosomes were isolated by ultracentrifugation and CAF-derived exosomal microRNAs were screened using next-generation sequencing technology. MiR-500a-5p expression was assessed by quantitative real-time polymerase chain reaction (qRT-PCR) and *in situ* hybridization; Tumor cell proliferation was determined by MTT assays and three-dimensioned (3D) cultures, and tumor metastasis was determined by Transwell assays *in vitro*. *In vivo* assays were performed in a nude mouse subcutaneous xenograft model.

**Results:** We confirmed that CAF-derived exosomes significantly promoted the proliferation and metastasis of breast cancer cells. MiR-500a-5p was highly expressed in MDA-MB-231 and MCF7 cells treated with CAF-derived exosomes. The upregulation of miR-500a-5p was also confirmed in CAFs and CAF-derived exosomes. MiR-500a-5p was transferred from CAFs to the cancer cells, and subsequently promoted proliferation and metastasis by binding to ubiquitin-specific peptidase 28 (USP28).

**Conclusions:** The present study demonstrates that CAFs promote breast cancer progression and metastasis via exosomal miR-500a-5p and indicate that inhibiting CAF-derived miR-500a-5p is an alternative modality for the treatment of breast cancer.

## Introduction

As the most common cancer in women, breast cancer is the second leading cause of cancer-related mortality worldwide among women and represents a serious threat to women's health 1. Tumors are encircled by an extracellular matrix and stromal cells. These cellular and non-cellular components of the tumoral niche comprise the tumor microenvironment 2. The crosstalk between cancer cells and the tumor microenvironment is essential for tumor progression and metastasis 3, 4. Cancer-associated fibroblasts (CAFs) are a major cellular component of the tumor microenvironment in most solid cancers 5. Accumulating evidence indicates that CAFs play an important role in cancer initiation, progression, epithelial-to-mesenchymal transition (EMT), metastasis and therapy resistance by communication with cancer cells via exosomes 6-8.

Exosomes are small membranous vesicles with a particle size of 30-150 nm 9. These small extracellular vesicles are critical cellular communicators encapsulating a variety of proteins, lipid, mRNAs, microRNAs, and lncRNAs 10, 11. Among the exosome-carrying molecules, miRNAs have drawn much attention due to their basic features. MiRNAs in exosomes, can be taken up by neighboring or distant cells and subsequently promote oncogenic signaling by suppressing target mRNAs in recipient cells 12-14. To verify the potential of a CAF-derived exosomal miRNA for use as a diagnostic marker or a therapeutic target for cancer, a comprehensive understanding of its mechanistic role in cancer progression is required.

In this study, we isolated CAFs and normal fibroblasts (NFs) from patient tissues and identified the differential miRNA profiles of cancer cells treated with CAF-derived exosomes using next-generation sequencing. Our results demonstrated that miR-500a-5p was directly transferred from CAFs to breast cancer cells via exosomes. We also identified the molecular mechanisms by which exosomal miR-500a-5p modulated the malignant phenotypes in breast cancer cells.

## Results

### CAFs derived from breast cancer patients promote the proliferation and migration of breast cancer cell through exosome

**Figure 1:**
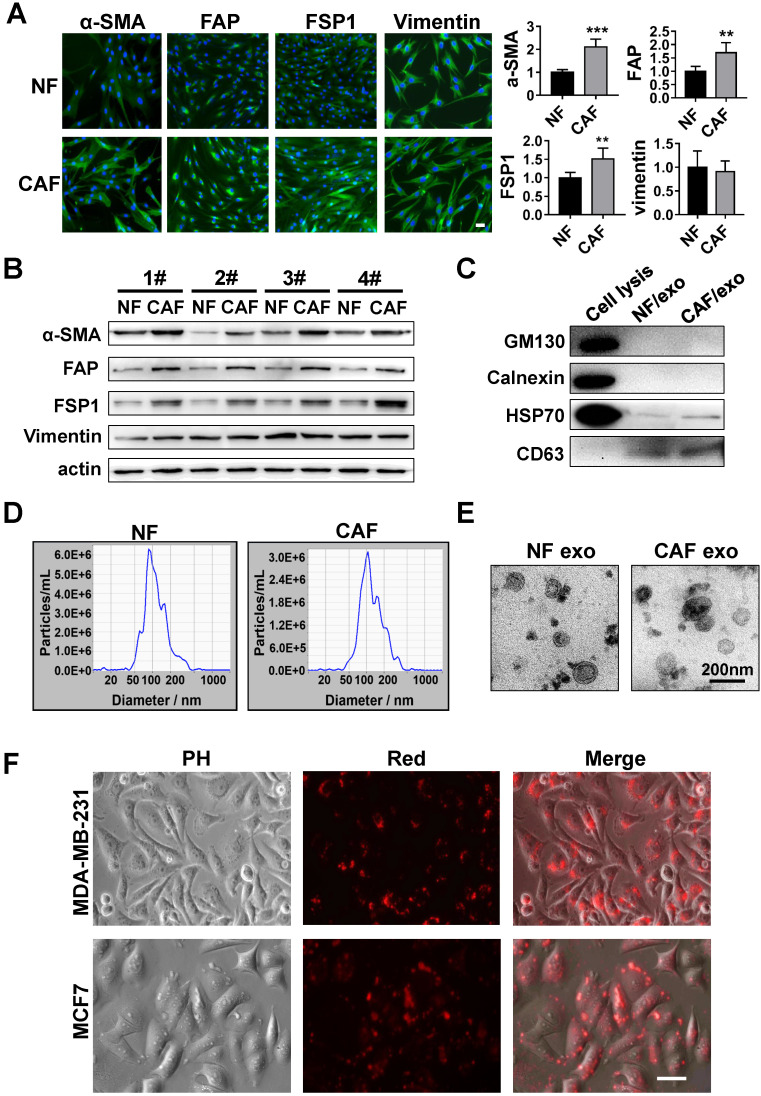
** Characteristics of CAFs derived from breast cancer patients and isolation of exosomes. (A)** Immunofluorescent staining and quantification of α-SMA, FAP, FSP1 and vimentin expression in NFs and CAFs (scale bar, 50 µm). **(B)** Western blot analysis of α-SMA, FAP, FSP1 and vimentin protein levels in four pairs of NFs and CAFs.** (C)** Western blot analysis of CD63, HSP70, calnexin and GM130 expression in exosomes isolated from NFs, CAFs and in cell lysates. **(D)** Nanoparticle tracking analysis of vesicles collected from NF- or CAF-conditioned medium. **(E)** Electron microscopy analysis of exosomes isolated from NF- or CAF-conditioned medium (scale bar, 200 nm). **(F)** Representative microscopy image showing the uptake of PKH26-labeled exosomes by MCF7 and MDA-MB-231 cells. Three independent experiments were performed and representative images are shown. Data are presented as means ± SEM (^**^P < 0.01; ^***^P < 0.001; Student's t-test).

CAFs and NFs were isolated from the breast carcinomas and adjacent normal breast tissue, respectively. The isolated CAFs and NFs possessed a spindle-shaped morphology and were both vimentin-positive. However, compared with NFs, CAFs expressed higher levels of the specific fibroblast markers α-smooth muscle actin (α-SMA), fibroblast activation protein (FAP), and fibroblast-specific protein 1 (FSP1) (Figure 1A-B). Exosomes, which are nanometric membrane vesicles secreted by both CAFs and NFs, play an important role in the communication between cells in the tumor microenvironment 10. We isolated exosomes from the conditioned media (CM) collected from cultures of CAFs and NFs by ultracentrifugation. Exosomes were characterized and quantified by Western blot analysis, qNano and electron microscopy. Western blot analysis confirmed the expression of the exosomal markers CD63 and HSP70, while GM130 (cis-Golgi compartment-specific marker) and calnexin (endoplasmic reticulum membrane marker) expression was absent (Figure 1C), which was consistent with previous reports on exosomes 13. Electron microscopy and qNano analysis confirmed the double-layer membrane nature and size of the exosomes (10-210 nm diameter) (Figure 1D, E). Furthermore, we evaluated uptake of these CAF-derived exosomes by the breast cancer cells using the fluorescent dye PKH26. MDA-MB-231 or MCF7 cells were cultured with CAF-derived exosomes labeled with PKH26. After 3 h, the presence of red fluorescence in the breast cancer cells indicated efficient uptake of the CAF-derived exosomes (Figure 1F).

**Figure 2:**
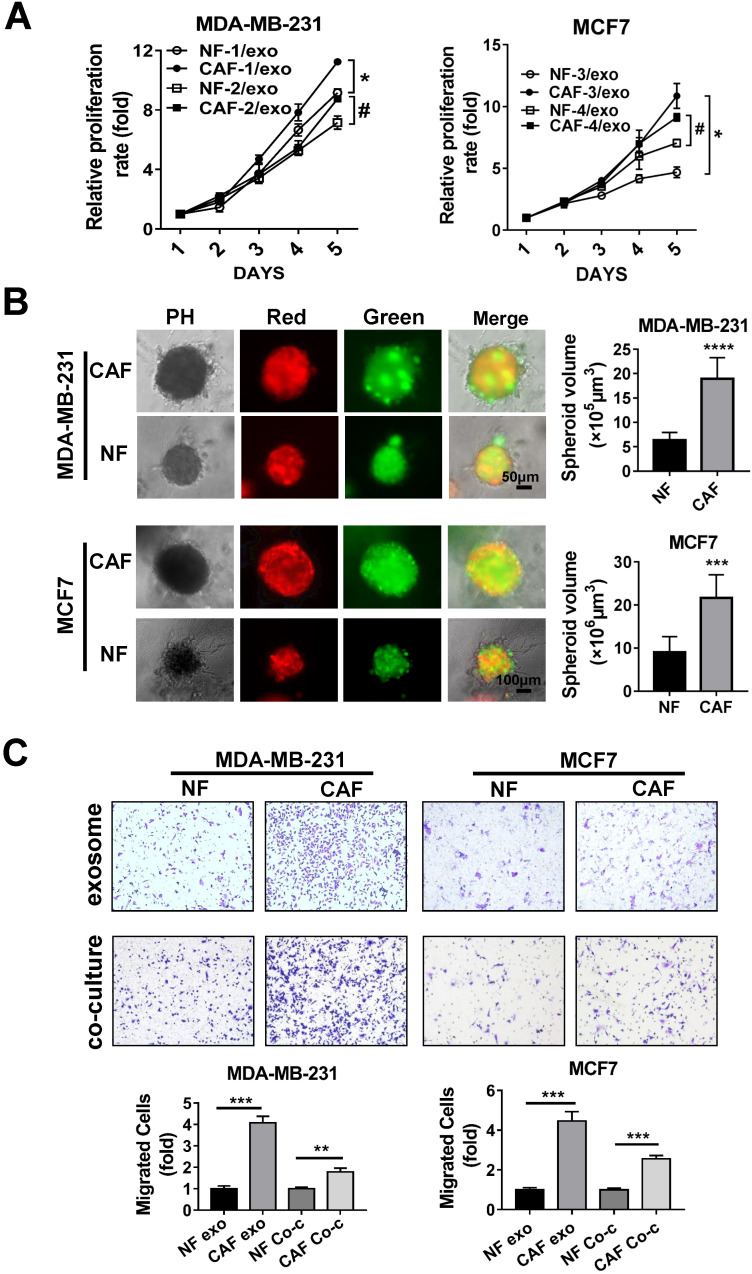
** CAF promotes the proliferation and migration of breast cancer cell through exosome. (A)** The relative proliferation rate was analyzed in MTT assays of MDA-MB-231 and MCF7 cells after incubation with NF- or CAF-derived exosomes. **(B)** 3D embedded assays of GFP-labeled MDA-MB-231 or MCF7 cells and mCherry-labeled CAFs or NFs. **(C)** MDA-MB-231 and MCF7 cells were incubated with NF- or CAF-derived exosomes or co-cultured with NFs or CAFs using Boyden chambers (4 µm pore size). Transwell assays were performed to detect the cell migration. Three independent experiments were performed and data are presented as means ± SEM (^#,*^P < 0.05; ^**^P < 0.01; ^***^P < 0.001; Student's t-test).

To confirm the impact of our patient-derived CAFs on tumor cell proliferation, MDA-MB-231 or MCF7 cells were cultured with CAF- or NF-derived exosomes and tumor cell proliferation was evaluated by MTT assay. Compared to the NF-derived exosomes, CAF-derived exosomes significantly promoted the proliferation of the breast cancer cells (Figure 2A). Meanwhile, by using the EV-free supernatant after ultracentrifugation, we found that CAF-mediated promotion of breast cancer cell proliferation and migration is in an exosome-dependent manner or, alternatively, the effect is not the result of co-purifying molecular complexes in the ultracentrifuged pellet (Figure S1). We further performed the three-dimensional (3D) embedded assays to further investigate the impact of CAFs on tumor cells. Green fluorescent protein (GFP)-labeled MDA-MB-231 or MCF7 cells and mCherry-labeled NFs or CAFs were mixed and micro-injected into semi-solidified Matrigel to culture 3D spheroids for 7 days. The observation of both red and green fluorescence suggested the spheroids consisted of fibroblasts and breast cancer cells. Under co-culture conditions, we measured the size of the spheroids and found that the spheroids containing CAFs exhibited a larger volume (Figure 2B).

Furthermore, in Transwell assays to verify the impact on the migration of breast cancer cells, we found that both CAF-derived exosomes and co-culture with CAF cells increased metastasis of MDA-MB-231 and MCF7 cells (Figure 2C). Thus, these data indicated that exosomes released by patient-derived CAFs accelerate the proliferation and migration of breast cancer cells.

### MiR-500a-5p is upregulated in CAF-derived exosomes

**Figure 3:**
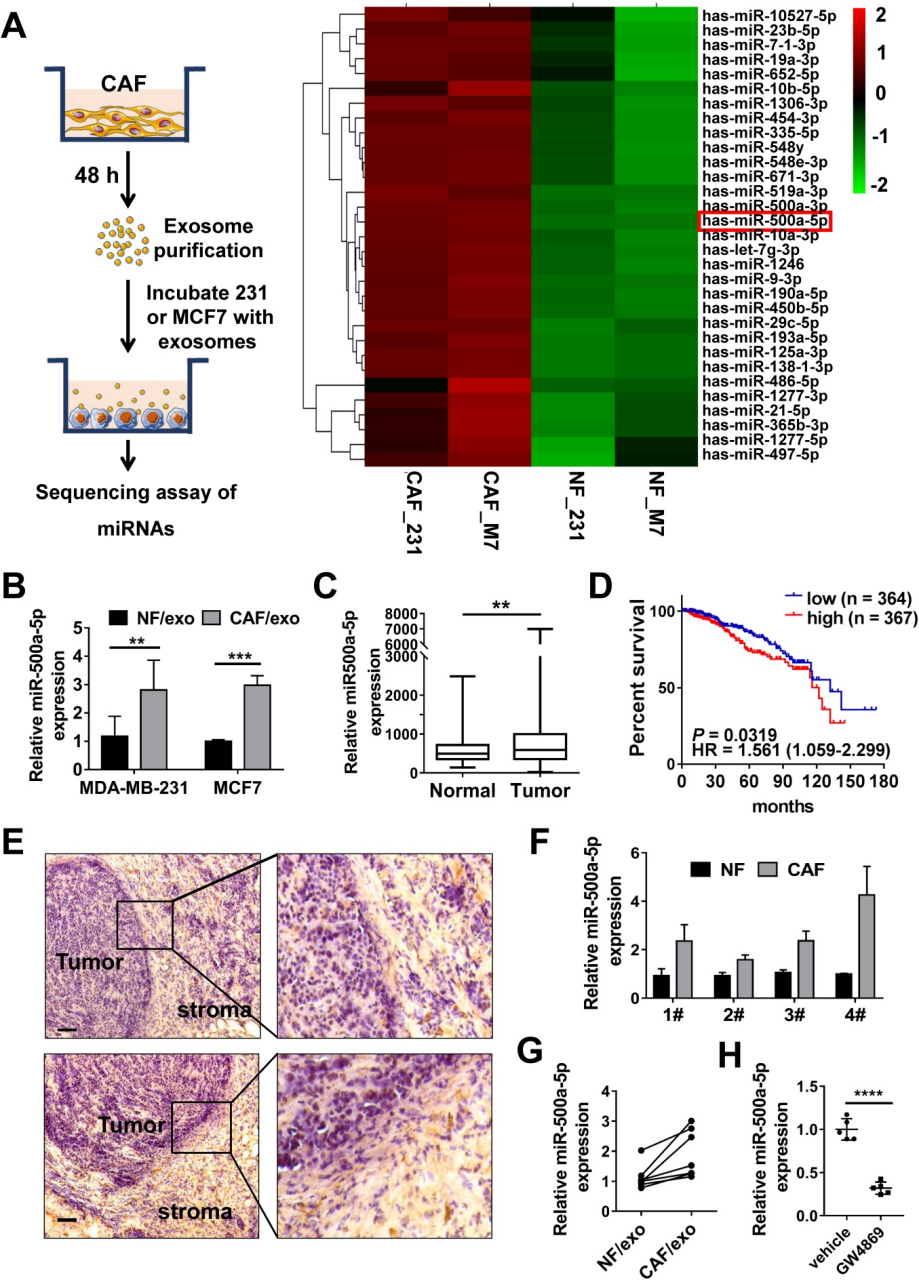
** MiR-500a-5p is upregulated in CAF-derived exosomes. (A)** Schematic (left) illustration of the co-culture system; the heat map (right) shows the expression of 31 miRNAs dysregulated in breast cancer cell lines co-cultured with exosomes from CAFs or NFs. **(B)** The relative expression of miR-500a-5p in MDA-MB-231 and MCF7 cells treated with CAF-derived exosomes. **(C)** Expression of miR-500a-5p in tumor tissues and normal tissues according to TCGA dataset (N: n = 103, T: n = 1,093). **(D)** Kaplan-Meier analysis showing the association between miR-500a-5p expression and overall survival in breast cancer patients (n = 733, P = 0.0211). **(E)** *In situ* hybridization (ISH) of miR-500a-5p in breast cancer tissues (scale bar, 100 µm). **(F)** qRT-PCR showing the expression of miR-500a-5p in paired CAFs and NFs.** (G)** qRT-PCR showing the expression of miR-500a-5p in exosomes derived from paired CAFs and NFs. **(H)** The effects of the EV secretion inhibitor GW4689 (10 mM) on exosome-dependent miRNA delivery from CAFs into MDA-MB-231 recipient cells. Data are presented as means ± SEM (^**^P < 0.01; ^***^P < 0.001; ^****^P < 0.0001; Student's t-test).

MiRNAs are frequently encapsulated in exosomes and involved in intercellular communication. To identify miRNAs that are transferred to breast cancer cells by exosomes secreted from CAFs, we incubated MDA-MB-231 and MCF7 cells with NF- or CAF-derived exosomes and further analyzed the miRNA profiles using miRNA microarrays. Data analyses identified that 31 miRNAs were up-regulated at least 1.5-fold. Among the 31 differentially-expressed miRNAs, miR-500a-5p was most increased (more than 4-fold) both in MDA-MB-231 cells and MCF7 cells incubated with CAF-derived exosomes (Figure 3A). These results were confirmed by qRT-PCR (Figure 3B). MiR-500a-5p is a less well-studied miRNA in breast cancer. By excavating high-throughput RNA-seq data deposited in The Cancer Genome Atlas (TCGA), a trend towards increased miR-500a-5p expression in breast cancer tissues was observed (Figure 3C). Moreover, the higher expression of miR-500a-5p contributed to a trend of poorer overall survival outcome in all types of breast cancer (Figure 3D). These data suggested that miR-500a-5p may have a certain cancer-promoting effect.

To explore the origin of miR-500a-5p in breast cancer, we assessed its expression in frozen tissue sections from breast cancer patients by *in situ* hybridization (ISH). Intriguingly, although miR-500a-5p expression was detected in both the cancer nest and tumor stroma, it was more highly expressed in the stroma (Figure 3E). To further confirm this pattern, we detected miR-500a-5p levels in isolated CAFs and NFs. As shown in Figure 3F, a significant increase in miR-500a-5p expression was detected in CAFs (Figure 3F). Furthermore, miR-500a-5p was upregulated in exosomes isolated from CAFs compared to those derived from NFs (Figure 3G). Importantly, prior addition (24 h) of inhibitor GW4869 to blocked CAFs exosome production decreased miR-500a-5p expression in MDA-MB-231 cells treated with CAFs-exosome (Figure 3H), suggesting that CAFs secrete extracellular miRNAs predominantly in an exosome-dependent manner.

### MiR-500a-5p transfection of CAFs increases exosomal miR-500a-5p levels and promotes breast cancer progression

**Figure 4:**
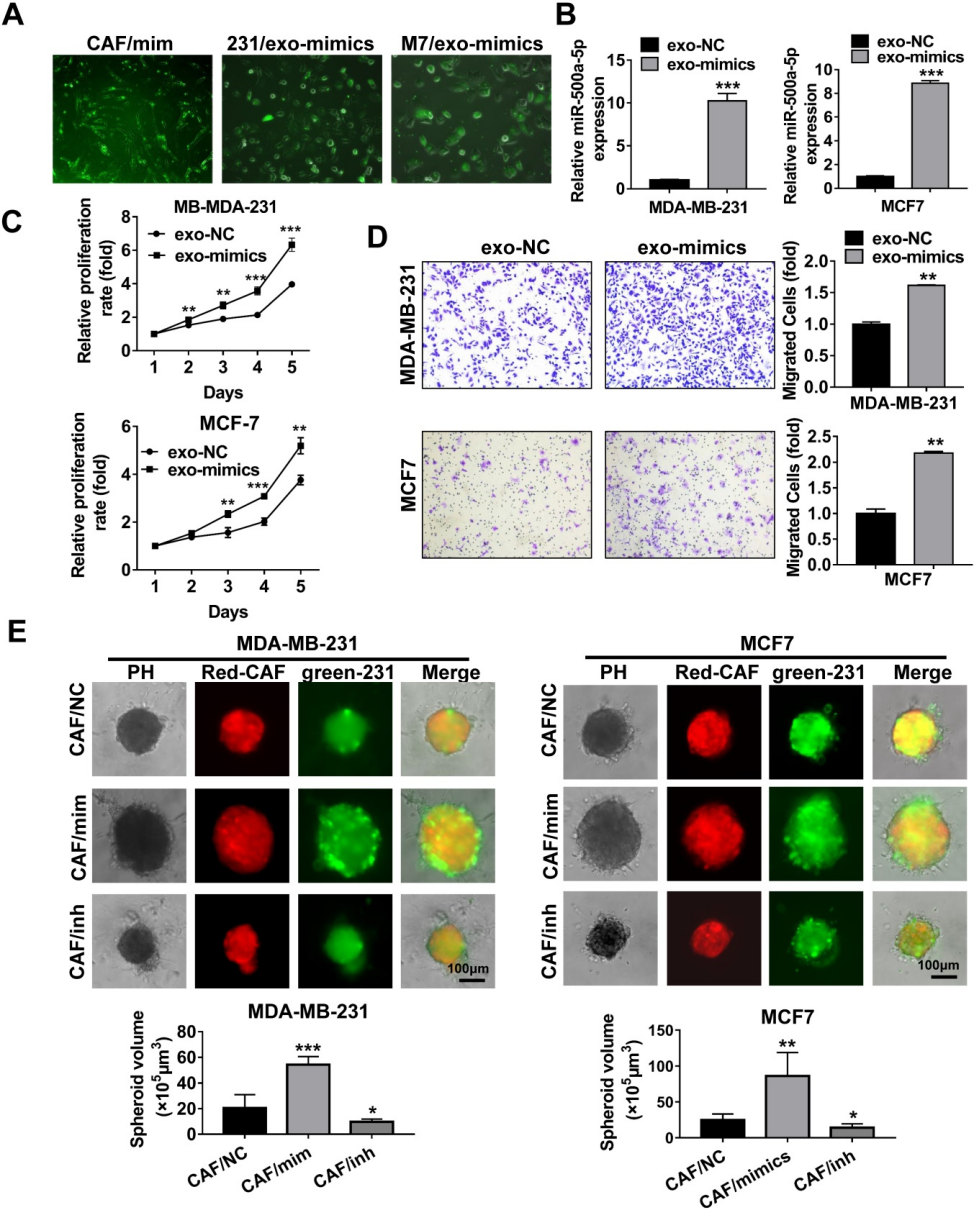
** MiR-500a-5p upregulation in CAFs promotes cancer cell progression. (A-D)** Exosomes was collected from CAFs transfected with FAM-tagged miR-500a-5p mimics and added to MDA-MB-231 and MCF7 cells. **(A)** Green fluorescence was observed by fluorescence microscopy. **(B)** qRT-PCR analysis of miR-500a-5p expression in MDA-MB-231 and MCF7 cells. **(C)** Cell proliferation was examined by MTT assay. **(D)** Transwell assays were utilized to assess cell migratory and invasive ability. **(E)** CAFs transiently transfected with miR-500a-5p mimics, inhibitor or negative control were 3D-cultured with MDA-MB-231 or MCF7 cells to observe cell spheroid formation ability. Three independent experiments were performed and data are presented as means ± SEM (^*^P < 0.05; ^**^P < 0.01; ^***^P < 0.001; Student's t-test).

We next investigated the ability of breast cancer cells to take up miR-500a-5p from exosomes secreted by CAFs. Exosomes from CAFs that were transiently transfected with FAM-tagged miR-500a-5p mimics were incubated with MDA-MB-231 and MCF7 cells. Evaluation by fluorescence microscopy revealed green fluorescence in MDA-MB-231 and MCF7 cells (Figure 4A). To determine whether the high level of miR-500a-5p in CAFs can be transferred to breast cancer cells, we incubated MDA-MB-231 and MCF7 cells with exosomes isolated from CAFs transfected with miR-500a-5p mimics or negative control (NC). qRT-PCR analysis indicated that miR-500a-5p expression was significantly upregulated in both cell lines following incubation with the CAF-derived exosomes (Figure 4B).

Next, we incubated MDA-MB-231 and MCF7 cells with exosomes isolated from CAFs overexpressing miR-500a-5p. MTT and Transwell assays showed that CAF-derived exosomal miR-500a-5p significantly promoted cell proliferation and migration (Figure 4C-D). Furthermore, mCherry-tagged CAFs transfected with miR-NC, miR-500a-5p mimics or miR-500a-5p inhibitor were mixed with GFP-labeled breast cancer cells and micro-injected into the semi-solidified Matrigel to culture 3D spheroids. Under co-culture conditions, the spheroids formed in the miR-500a-5p overexpressing group were significantly larger than those in the NC group, whereas smaller spheroids were formed in the miR-500a-5p inhibition group (Figure 4E).

### MiR-500a-5p promotes proliferation and metastasis of breast cancer cells

**Figure 5:**
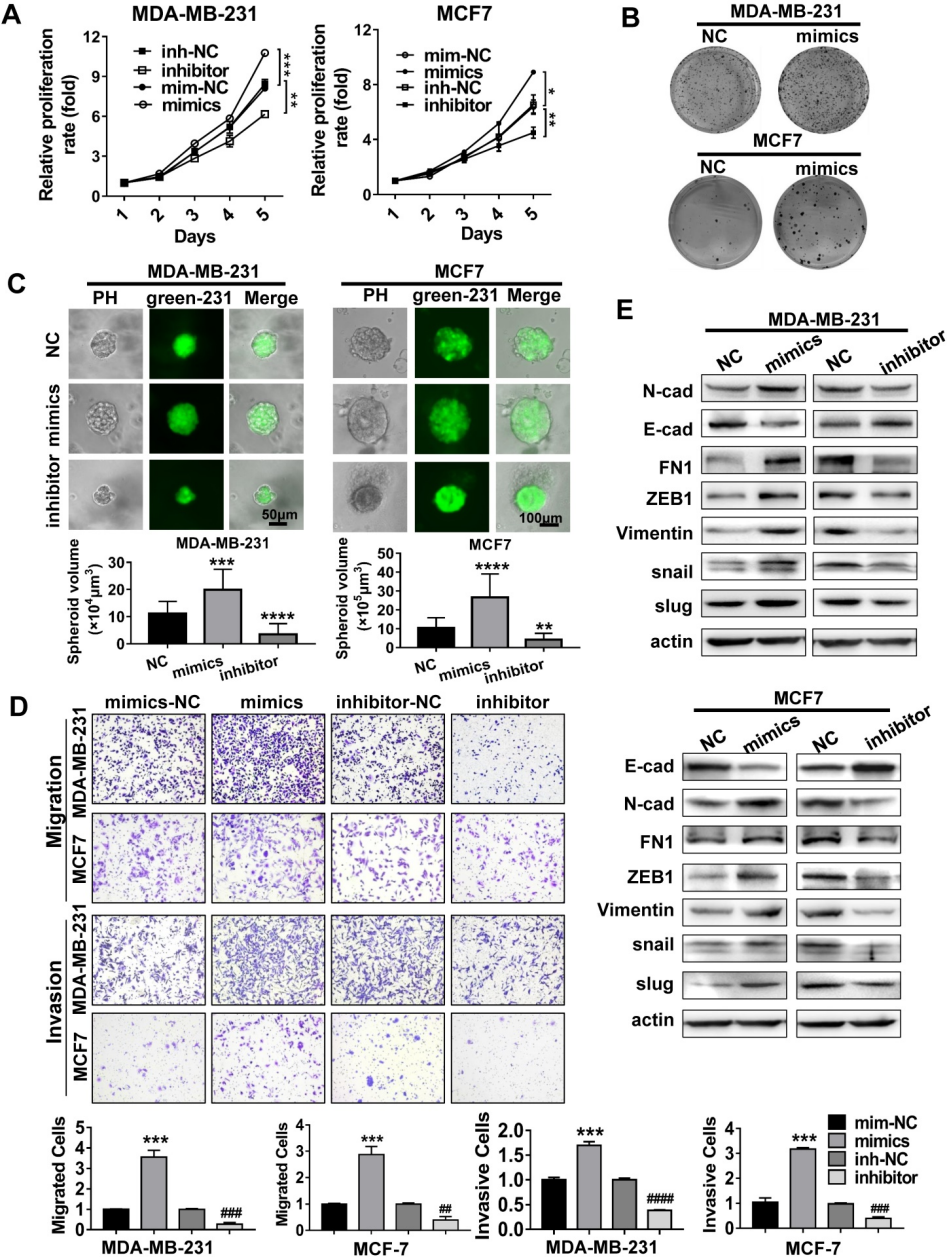
** MiR-500a-5p promotes proliferation and metastasis of breast cancer cells. (A-E)** MiR-500a-5p was overexpressed and inhibited in MDA-MB-231 and MCF7 cells.** (A)** Cell proliferation, **(B)** colony formation, **(C)** 3D spheroid formation, and **(D)** migratory and invasive abilities were evaluated by MTT, colony formation, 3D culture, and Transwell assays, respectively. **(E)** Western blot analysis of the expression of FN1, ZEB1, N-cad, E-cad, vimentin, snail, and slug in the indicated cells. β-Actin is a loading control. Data are presented as means ± SEM (^*^P < 0.05;^ ##, **^P < 0.01; ^###, ***^ P < 0.001; Student's t-test).

We next investigated the biological functions of miR-500a-5p in breast cancer cells. MDA-MB-231 cells and MCF7 cells were transiently transfected with miR-500a-5p mimics or miR-500a-5p inhibitor. MTT and the colony formation assays revealed that miR-500a-5p promoted the proliferation and colony formation ability of both cell types compared with the NC group, while these effects were reversed following miR-500a-5p inhibition (Figure 5A-B). Furthermore, GFP-tagged MDA-MB-231 cells transfected with miR-NC, miR-500a-5p mimics or inhibitor were used to culture 3D spheroids. Evaluation of green fluorescence showed that cells transfected with miR-500a-5p mimics formed larger spheroids compared with the control group, whereas those transfected with miR-500a-5p inhibitor formed small spheroids (Figure 5C). These findings suggested the positive effect of miR-500a-5p on breast cancer cell proliferation. Furthermore, we performed Transwell and scratch wound healing assays to analyze the effect of miR-500a-5p on tumor metastasis (Figure 5D, S2A-C). The results indicated that miR-500a-5p enhanced both the migration and invasive abilities of MDA-MB-231 and MCF7 cells, while both functions were significantly reduced after miR-500a-5p inhibition. Given that EMT plays a vital role in cancer metastasis, we further investigated the expression of EMT-related markers after ectopic expression and depletion of miR-500a-5p. Western blot analysis demonstrated that ectopic expression of miR-500a-5p decreased the expression of epithelial markers and increased the expression of mesenchymal markers (Figure 5E, S4A), highlighting the significant role of miR-500a-5p in regulating EMT in breast cancer cells. Therefore, these results demonstrated that miR-500a-5p promotes cell proliferation, migration, invasion, and EMT in breast cancer cells.

### USP28 is a direct target gene of miR-500a-5p

**Figure 6:**
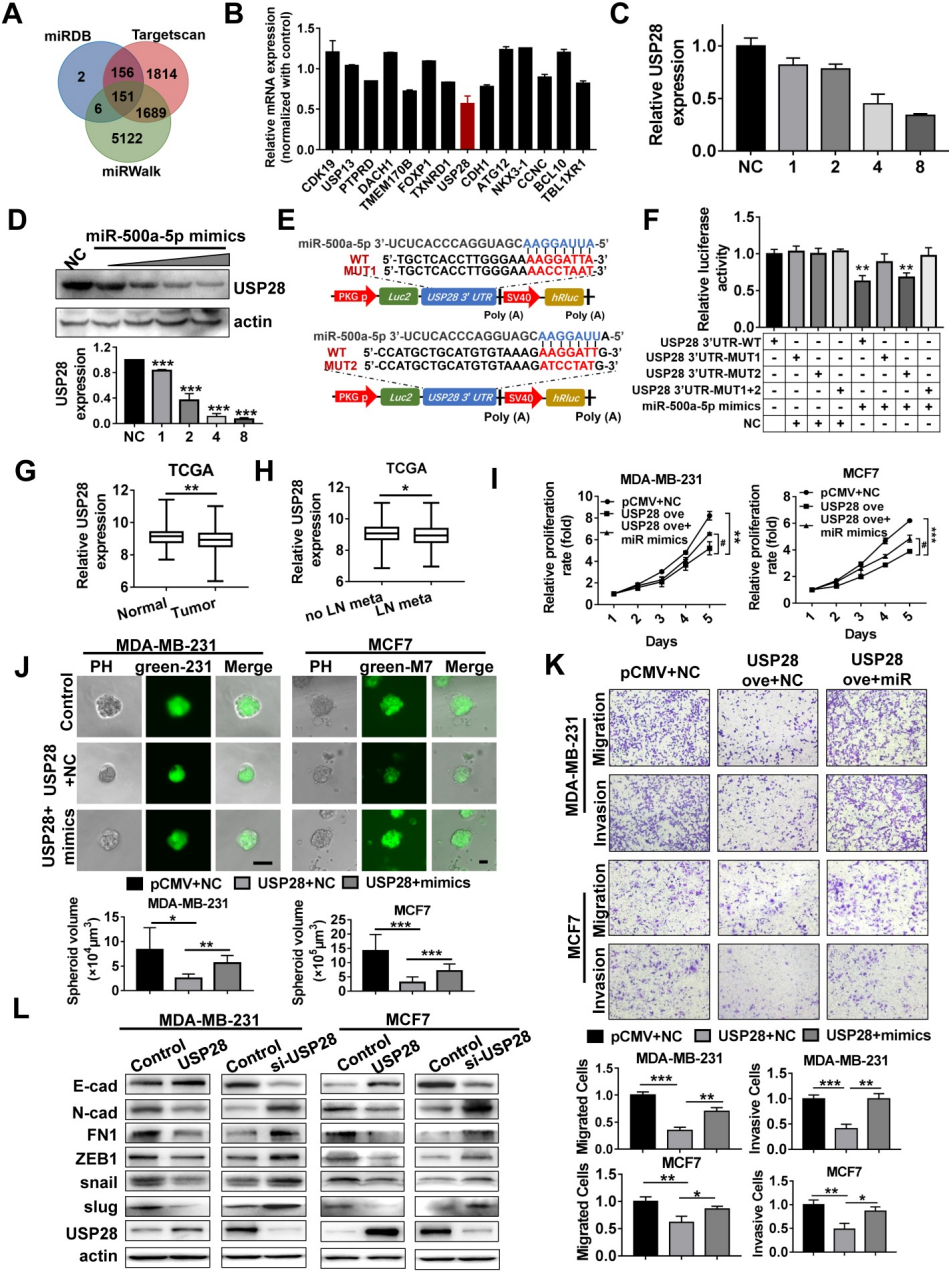
** USP28 is a direct target gene of miR-500a-5p. (A)** Venn diagram illustrates the putative candidate target genes of miR-500a-5p predicted by miRDB, TargetScan, and miRWalk. **(B)** Expression of the candidate miRNAs after miR-500a-5p overexpression in MCF7 cells was assessed by qRT-PCR. **(C)** qRT-PCR and **(D)** Western blot assays showing that USP28 expression is regulated by miR-500a-5p in a dose-dependent manner. 231 cells (about 5×10^6^/group) were transfected with NC or gradient miR-500a-5p mimics. The dose “1” represents 0.16 µM. **(E)** Schematic diagram of luciferase reporter plasmid constructed containing wild-type (WT) and mutant (MUT) binding sites in the USP28 3'-UTR. **(F)** The luciferase activity of WT or MUT plasmids after co-transfection with miR-500a-5p mimics into HEK-293T cells. **(G)** The USP28 mRNA expression level in TCGA dataset in normal and tumor tissues. **(H)** Comparison of the USP28 expression in breast cancer patients with or without lymph node metastasis in TCGA dataset. **(I)** Cell proliferation was detected by MTT assay in MDA-MB-231 and MCF7 cells with USP28 overexpression combined with or without miR-500a-5p mimics. **(J)** Spheroid formation by MDA-MB-231 cells with USP28 overexpression combined with or without miR-500a-5p mimics.** (K)** Cell migration and invasion of MDA-MB-231 and MCF7 cells detected by Transwell assay. **(L)** Western blot assays of the expression of E-cad, N-cad, FN1, ZEB1, snail, slug, and USP28 in MDA-MB-231 and MCF7 cells with USP28 overexpression and inhibition. β-Actin is a loading control. Data are presented as means ± SEM (^#, *^P < 0.05;^ **^P < 0.01; ^***^P < 0.001; Student's t-test).

To explore the mechanism by which miR-500a-5p promotes cancer progression in breast cancer cells, three miRNA target gene databases (miRDB, TargetScan and miRWalk) were searched to identify the potential target of miR-500a-5p. In total, 151 genes were listed in all three algorithms (Figure 6A). Among all the predicted genes, we chose the 14 most highly ranked genes as candidates. As the most downregulated gene according to the qRT-PCR analysis, USP28 was selected for further investigation (Figure 6B). USP28 expression in MDA-MB-231 cells was reduced following miR-500a-5p mimics transfection in a dose-dependent manner at both the mRNA and protein levels (Figure 6C-D). MRE analysis suggested that miR-500a-5p has two potential binding sites within the 3'-UTR of USP28 (Figure 6E). To verify USP28 as a direct target of miR-500a-5p, wild-type or mutated binding sites in the 3'-UTR of USP28 were cloned into the luciferase reporter plasmid to generate the wild-type (WT) and mutant luciferase reporters (MUT1 or MUT2 or MUT1+2). After co-transfection with miR-500a-5p mimics, we observed reduction in luciferase activity in both the WT and MUT2 groups, while there was no diminution in luciferase activity in the MUT1 and MUT1+2 groups (Figure 6F). These findings suggested that MUT1 contained the affirmative mutant binding sites in the 3'-UTR of USP28, thus indicating that USP28 is a direct target of miR-500a-5p.

Furthermore, gene expression profiles of breast cancer from TCGA showed that the expression of USP28 had a downward trend in tumor tissues (Figure 6G). Moreover, USP28 also showed a trend towards reduced expression in breast cancer patients with lymph node metastasis (Figure 6H). These findings suggested that USP28 may function as a breast cancer suppressor which was in contrast to the function of miR-500a-5p.

Given that our findings indicated that USP28 is regulated by miR-500a-5p, gain- and loss- of function experiments were conducted to determine the biological characteristics of USP28 in breast cancer cells. MTT assays showed that USP28 overexpression reduced cell proliferation in MDA-MB-231 and MCF7 cells, and these effects were partially reversed by transfection with miR-500a-5p mimics (Figure 6I). We also conducted 3D cultures to investigate the roles of miR-500a-5p and USP28 in cancer cell proliferation. Ectopic USP28 expression in MDA-MB-231 and MCF7 cells resulted in the formation of small spheroids and this effect was antagonized by miR-500a-5p overexpression (Figure 6J). Transwell assays showed that USP28 overexpression reduced both cell migration and invasion in MDA-MB-231 and MCF7 cells, and these effects were partially reversed by transfection with miR-500a-5p mimics (Figure 6K). Next, we designed three small interfering RNAs (siRNAs) to inhibit USP28 expression and chose the siRNA with the best interfering effect for the MTT and Transwell assays (Figure S3A). The results showed that cell proliferation, migration and invasion were enhanced after USP28 inhibition (Figure S3B-C).

We then detected the expression of EMT-related markers in breast cancer cells after upregulation and downregulation of USP28. Following USP28 overexpression in MCF7 and MDA-MB-231 cells, E-cadherin expression was significantly increased, while expression of N-cad, FN1, ZEB1, snail and slug was reduced. USP28 knockdown showed converse patterns of expression of these EMT markers (Figure 6L, S4B). Therefore, these data indicated that miR-500a-5p promotes breast cancer progression and metastasis by sponging USP28.

### CAFs with upregulated miR-500a-5p promote breast cancer progression in a xenograft animal model

**Figure 7:**
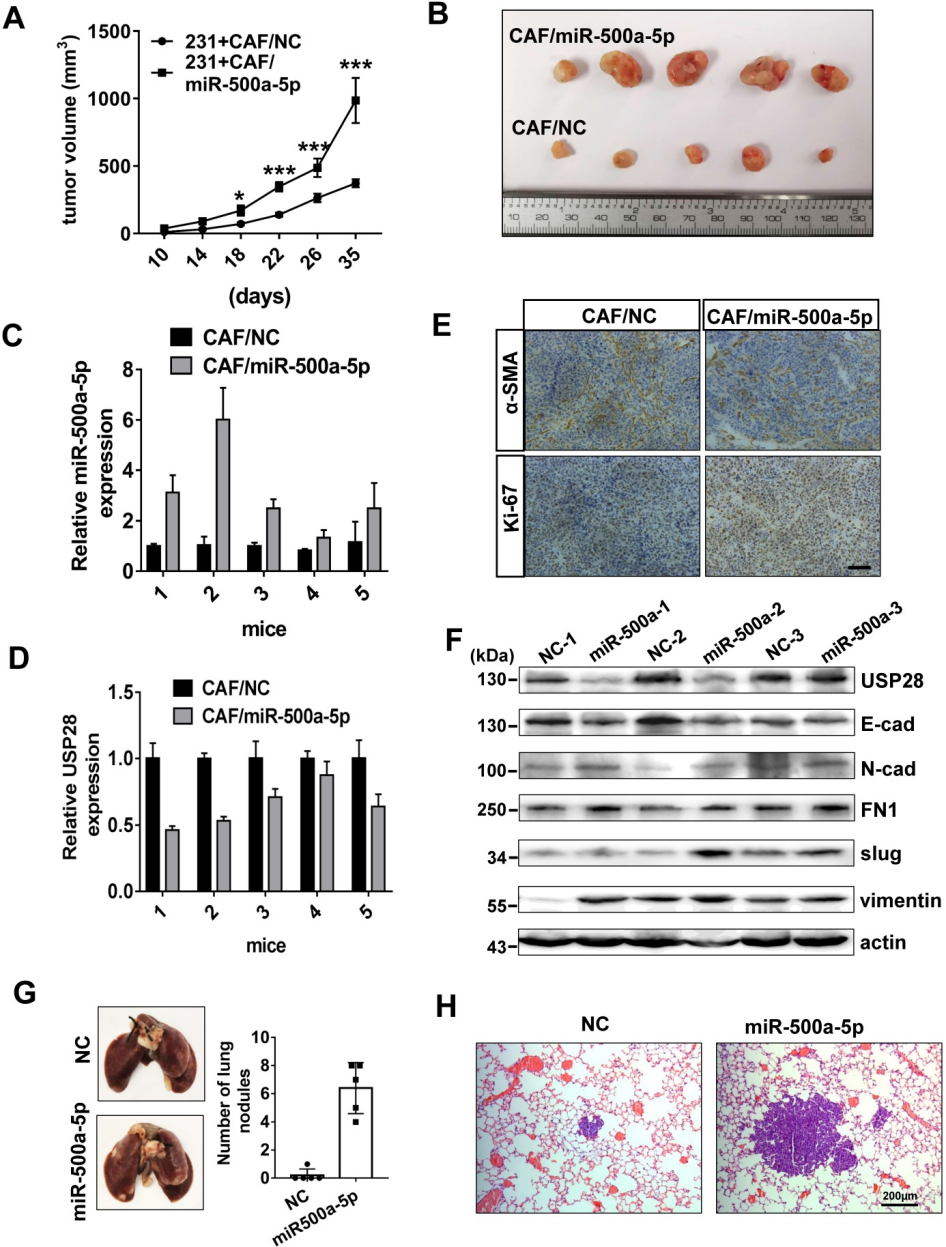
** CAFs with upregulated miR-500a-5p expression promotes breast cancer progression in a xenograft animal model. (A-F)** MDA-MB-231 cells were mixed with CAFs/miR-500a-5p or CAFs/NC, and subcutaneously injected into nude mice. **(A)** The tumor volumes were measured every 4 days from 10 days after the injection. Data are presented as means ± SEM (n = 5,^ *^P < 0.05;^ ***^P < 0.001; Student's t-test). **(B)** Photographs illustrating xenografted tumors. **(C)** qRT-PCR showing the expression of miR-500a-5p in tumor tissues. **(D)** qRT-PCR showing the expression of USP28 in tumor tissues. **(E)** IHC staining of α-SMA and Ki-67 in xenografted tumors (scale bar, 100 µm). **(F)** Western blot showing the expression of USP28 and the EMT markers in the indicated tumor tissues. β-Actin is utilized as loading control. **(G)** Images and quantification of pulmonary surface nodules after tail vein injection of NC or miR-500a-5p overexpressing MDA-MB-231 cells. **(H)** H&E-stained lung section images, scale bar: 200 µm.

**Figure 8:**
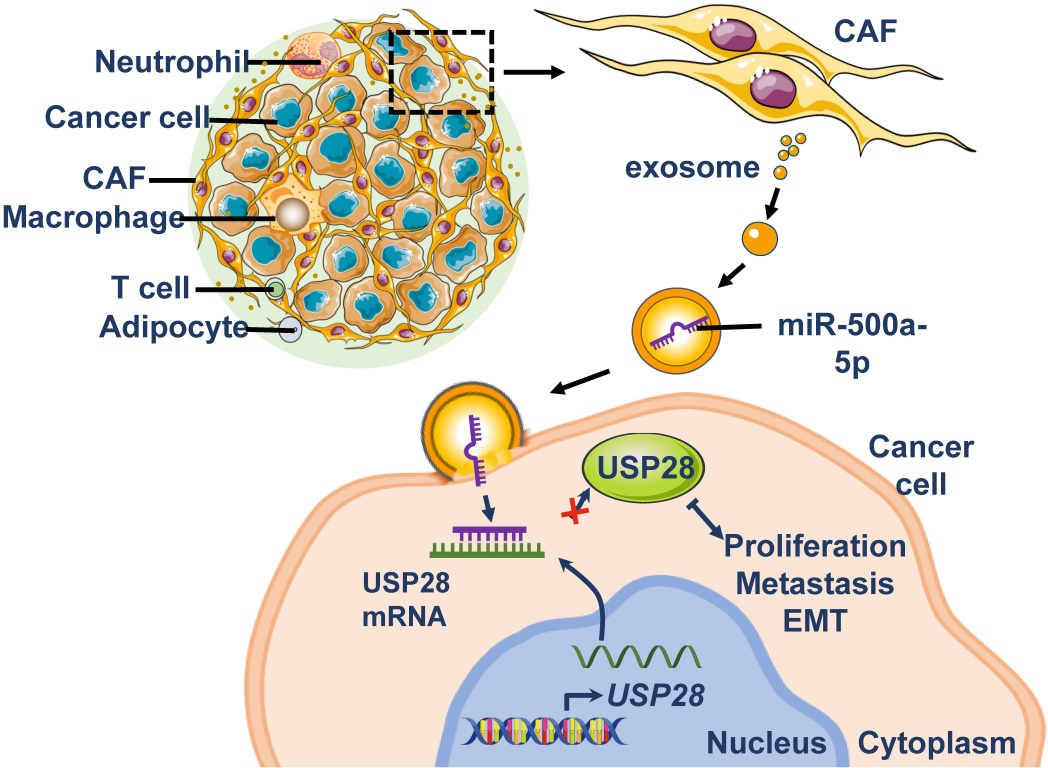
** Schematic representation of the proposed mechanism.** MiR-500a-5p in CAFs is transferred into tumor cells via exosomes and promotes cell proliferation, metastasis, and EMT via the miR-500a-5p/USP28 axis.

To evaluate the effect of miR-500a-5p on breast cancer *in vivo*, we generated CAFs stably expressing miR-500a-5p by lentivirus infection. Then, MDA-MB-231 cells were co-injected nude mice with miR-500a-5p-expressing CAFs (CAF/miR-500a-5p) or negative control CAFs (CAF/NC). We found that tumor volume and size were significantly larger in the CAF/miR-500a-5p group (Figure 7A-B). qRT-PCR revealed that miR-500a-5p expression was higher in the CAF/miR-500a-5p group, whereas USP28 showed the opposite expression pattern (Figure 7C-D). Next, IHC analysis confirmed α-SMA expression in both groups and stronger Ki67 expression in the CAF/miR-500a-5p group (Figure 7E). These results suggested the involvement of CAFs in the xenograft tumor composition and indicated that miR-500a-5p increases breast cancer cell proliferation* in vivo*. Moreover, examination of EMT markers in the tumors by Western blot analysis showed a positive correlation of high miR-500a-5p expression with low levels of USP28, E-cad, and high levels of N-cad, FN, slug and vimentin (Figure 7F). To determine the effects of miR-500a-5p expression on breast cancer cell metastasis *in vivo*, MDA-MB-231 cells stably expressing miR-320a or NC were injected into nude mice intravenously. Lungs were harvested 6 weeks after the cell injections, and we found that miR-500a-5p significantly increased surface lung metastases and the lung metastatic burden (Figure 7G-H). Collectively, our data suggested that high levels of miR-500a-5p in CAFs are transferred into tumor cells and downregulates USP28 expression, which promotes cell proliferation, metastasis and EMT in breast cancer (Figure 8).

## Discussion

In recent decades, the roles of the microenvironment in cancer progression, metastasis and therapeutic outcome have gained increasing attention 4, 15. CAFs have emerged as key players among stromal cells, owing to their abundance in most solid tumors and their diverse tumor-restraining/promoting roles 16-19. In this study, we isolated patient-derived breast CAFs and confirmed their tumor-promoting roles by comparison with NFs. Previous studies demonstrated that CAFs and cancer cells communicate not only through classical paracrine signaling mechanisms (cytokines, chemokines) 20-22, but also through exosomes 10, 23. Exosomes are endosome-derived microvesicles that contain lipids, proteins and nucleic acids (DNA, mRNA or non-coding RNAs, such as miRNAs and lncRNAs) 10. As the major RNA component of exosomes, miRNAs are dysregulated in several types of cancers 24 and modulate cancer progression and metastasis 25, 26. Exosomal transfer of several miRNAs, such as miR-196a, miR-21, and miR-1228, have been shown to play an important role in communication between cancer cells and the tumor microenvironment 12, 13, 27, 28.

In this study, we identified miR-500a-5p as a CAF-derived exosomal cargo that promoted the proliferation and metastasis of breast cancer cells. Previous studies have shown that miR-500a-5p (known as miR-500a) was upregulated in hepatocellular carcinoma and gastric cancer tissues and promoted tumor progression and metastasis 29-32. In contrast, miR-500a-5p was downregulated in colorectal cancer tissues and remarkably inhibited colorectal cancer cell proliferation and migration by targeting HDAC2 33. MiR-500a-5p is a less well-studied miRNA in breast cancer. Although Esposti et al. suggested that miR-500a-5p regulated oxidative stress response genes in breast cancer and predicted cancer survival 34, the roles of miR-500a-5p in breast cancer and the underlying mechanisms have not yet been elucidated. In our study, ISH revealed that miR-500a-5p is significantly overexpressed in the stroma compared with the levels detected in the tumor parenchyma. By incubating breast cancer cells with exosomes isolated from patient-derived CAFs, we confirmed that miR-500a-5p was transferred to the cancer cells and increased their proliferation and metastasis *in vitro* and *in vivo*.

MiRNAs are a family of short (20-24 nt) non-coding RNAs that post-transcriptionally regulate gene expression by affecting both the stability and translation of mRNAs in multicellular organisms 35. As one of the most prevalent and important post-translational modifications, ubiquitination is engaged in multiple cancer-related pathways 36. The deubiquitylating enzymes (DUBs) are involved in cancer-modulated process by regulating the ubiquitination process. In this study, the DUB USP28 was verified as a target of miR-500a-5p. USP28 has been shown to play complex and versatile modulatory roles in various cancers 36, through its involvement in multiple pathways that can have opposing functions. Previous studies revealed that USP28 is required for the stability of oncoproteins such as c-MYC in colon cancer 37-39, and c-JUN and NOTCH in colorectal cancer 38. In addition, USP28 was found to stabilize the tumor suppressor Chk2. Notably, USP28 performs dual regulation of the proto-oncogenic FBW7. USP28 can counteract the activity of FBW7 and promote the stability of FBW7-substrates in cancer cells 40. In contrast, USP28 was also found to stabilize FBW7 directly, leading to a reduction in FBW7 substrate proteins 41. Thus, USP28 functions as either a tumor promotor or suppressor depending on the status of autocatalytic ubiquitination of FBW7 36. In breast cancer, Cao et al. found that HDAC5 positively regulated LSD1 by promoting the stability of USP28 protein, which is a confirmed deubiquitinase of LSD1. In addition, HDAC5 promoted breast cancer development and progression in a LSD1-dependent manner 42, however, there were no direct evidence that USP28 was involved in breast cancer progression in the study. In contrast, in a detailed study on the function of USP28 in breast cancer 43, Richter et al. found that USP28 deficiency in breast cancer cells enhances conversion toward a more aggressive phenotype by promoting EMT, proliferation, migration, angiogenesis, and decreased adhesion. Furthermore, a positive correlation between USP28 expression and survival was identified in patients with ductal breast carcinomas. However, the underlying mechanisms of breast cancer suppression by USP28 are not clear. In accordance with these findings, our study revealed that USP28, as the target gene of miR-500a-5p, reversed the ability of miR-500a-5p to promoted breast cancer cell proliferation and metastasis, indicating that USP28 functions as a breast cancer suppressor. Given the complexity of the various functions of USP28, specific mechanism for inhibiting breast cancer will be further studied in our future work.

In conclusion, our results show that patient-derived CAF exosomal miR500a-5p can confer an aggressive phenotype in breast cancer cells through its transfer from neighboring CAF cells. Thus, our findings suggest that inhibiting exosomal miR500a-5p is a new strategy for suppressing breast cancer growth and metastasis.

## Materials and methods

### Ethics statement

All the clinical samples were obtained from patients with invasive breast carcinoma without previous radiotherapy or neoadjuvant chemotherapy treated at the Qilu Hospital, Shandong University (Shandong, China). Our study was approved by the Ethics Committee of Qilu hospital, Shandong University (the ethical permission number: KYLL-2017(KS)-084). Written informed consent was provided by all participants prior to enrollment. All experimental methods were conducted in accordance with the Helsinki Declaration. All animal studies were undertaken in accordance with the NIH Guide for the Care and Use of Laboratory Animals.

### Isolation and culture of patient-derived fibroblasts

Primary CAFs and NFs were isolated from invasive ductal carcinoma samples obtained from patients undergoing breast ablation or resection. CAFs were derived from the area at the epicenter of the tumor with a maximum diameter of less than 10 mm, whereas NFs were obtained from the zone of normal tissue, more than 20 mm away from the tumor margin. Tissue samples were minced and digested for 2 h at 37 ºC with shaking in collagenase/hyaluronidase (#07912, Stem Cell Technologies, Durham, NC, USA) diluted in Dulbecco's modified Eagle's medium (DMEM; HyClone, Logan, UT, USA) supplemented with 10% fetal bovine serum (FBS; Sigma, St. Louis, MO, USA). After centrifugation, cell pellets were resuspended in DMEM supplemented with 10% FBS and filtered through a 100 μm cell strainer. The cells were then seeded into 60 mm tissue culture dishes and cultured in DMEM supplemented with 100 U/ml penicillin, 100 μg/ml streptomycin and 10% FBS at 37 ºC in a humidified atmosphere containing 5% CO_2_. NFs and CAFs were further identified by detection of the CAF-specific markers α-SMA, FAP and FSP1.

For conditioned medium preparation, the fibroblasts were incubated with DMEM containing 10% exosome-free FBS for 2 days. The conditioned medium was collected, centrifuged at 1,000 ×*g* for 5 min and 10,000 ×*g* for 30 min before being filtered (0.22-μm pore size).

For co-culture experiments, CAFs or NFs and cancer cells were mixed at a ratio of 3:1 by seeding cancer cells in six-well plates and then seeding CAFs or NFs in the upper chambers of Boyden chambers (4 μm pore size; Millipore, Darmstadt, Germany).

### Exosome isolation and fluorescent labeling

Exosomes were isolated from the conditioned medium by differential centrifugation as previously described 13. In brief, conditioned medium was centrifuged at 300 ×*g* for 10 min and then at 2,000 ×*g* for 20 min at 4 ºC to remove cells. The supernatant was then centrifuged at 10,000 ×*g* for 30 min at 4 ºC to remove cell debris. The exosomes in the supernatant were pelleted by ultracentrifugation at 100,000 ×*g* for 90 min and resuspended in PBS before collection by ultracentrifugation again at 100,000 ×*g* for 90 min. The exosome particle size was measured by nanoparticle tracking analysis (NTA) using ZetaView PMX 110 (Particle Metrix, Meerbusch, Germany). The exosomes were used for RNA/protein extraction or cell treatment.

Purified exosomes were labeled using the PKH26 red fluorescent labeling kit (Sigma) according to the manufacturer's protocol as previous studies 44, 45. In brief, the exosomes enriched by ultracentrifugation were resuspended in 100 μL of diluent C and mixed with 100 μL PKH26 dye solution (4 × 10^-6^ M). After incubated for 5 min, 200 μL of serum was added to stop. The labeled exosomes were then washed twice with PBS and coincubated with breast cancer cells. Uptake of labeled exosomes by breast cancer cells over 3 h was observed under a microscope.

### Transmission electron microscopy

Exosomes were dropped onto the copper grid and negatively stained with 2% phosphotungstic acid for 2 min. After air-drying, the samples were observed using a transmission electron microscope (FEI Tecnai G2 Spirit, Thermo Scientific, USA) at 80 kV.

### *In situ* hybridization

ISH was performed to determine the expression of miR-500a-5p using the Enhanced Sensitive ISH Detection Kit (POD) (MK1030, Boster Biological Technology, Wuhan, China) according to the manufacturer's instructions. In brief, frozen sections (10 mm thick) prepared from formalin-fixed tissues were digested with proteinase K at 37 ºC for 2 min. After prehybridization, slides were hybridized with miR500a-5p or scramble DIG-labeled probes at 37 ºC overnight followed by washing with SSC buffers. Subsequently, the slides were blocked and incubated with biotinylated mouse anti-DIG antibody and streptavidin-biotin-peroxidase complex (SABC) at 37 ºC, followed by three rinses with 0.5M phosphate-buffered saline (PBS). The sections were then incubated with avidin-biotin peroxidase complex. The results were visualized by development of a colored substrate 3,3'-diaminobenzidine (DAB) and counterstaining with hematoxylin. Finally, the sections were dehydrated in a graded ethanol series, cleared in xylene, and mounted with coverslips.

### Cell cultures and cell transfection

Breast cancer cell lines (MDA-MB-231 and MCF7) were purchased from American Type Culture Collection (ATCC, USA) and characterized by Genetic Testing Biotechnology Corporation (Suzhou, China). Cells were routinely cultured in the DMEM supplemented with 1% penicillin/streptomycin and 10% FBS at 37 ºC under 5% CO_2_. Cells were transfected using Lipofectamine 2000 reagent (Invitrogen, Carlsbad, CA, USA) according to the manufacturer's instructions.

### Plasmid construction, inhibitors and mimics

The cDNA of USP28 were cloned into the pcDNA3.1-Myc/His vector (Invitrogen) and verified by sequencing. For luciferase reporter assays, PCR-derived fragments from the 3′-UTR of USP28 (WT) was cloned into the pmiRGLO vector (Promega, Madison, WI, USA). The mutant luciferase reporters (MUT1 or MUT2 or MUT1+2) were generated by PCR-based site-directed mutagenesis. MiR-500a-5p mimics, inhibitors, USP28 siRNA and negative controls were purchased from GenePharma (Shanghai, China). The sequences were as follows (sense strand): miR-500a-5p mimics, 5'-UAAUCCUUGCUACCUGGGUGAGA-3'; NC, 5'-UUCUCCGAACGUGUCACGU-3'; miR-500a-5p inhibitors, 5'-UCUCACCCAGGUAGCAAGGAUUA-3'; Inhibitor NC: 5'-CAGUACUUUUGUGUAGUACAA-3'; USP28 siRNA, 5'-GGCCUAGAACUCUAUCAAA-3'.

### Immunofluorescence

Fibroblast cells grown on cover slips were fixed with 4% paraformaldehyde, permeabilized with 0.1% Triton X-100, blocked in 3% goat serum, and incubated overnight at 4 ºC with primary antibodies (α-SMA, CST, #19245, 1:500; FAP, Affinity Biosciences #AF5344, 1:1000; FSP1, Affinity Biosciences, #DF6516, 1:500; vimentin, Millipore, MAB3400, 1:1000). Thereafter, the samples were incubated with an Alexa Fluor 488-conjugated anti-Rabbit IgG (Invitrogen, USA; 1:500) at room temperature for 60 min in the dark. Nuclei were detected by treatment with 4′,6-diamidino-2-phenylindole (DAPI, Invitrogen, USA, 1:300).

### Western blot analysis

Cells were harvested and proteins were extracted from cells as previously described 46. The protein concentration was determined using a protein assay kit (Bio-Rad, Hercules, CA, USA). Equal amounts of proteins (30 µg) were resolved by sodium dodecyl sulfate polyacrylamide gel electrophoresis (SDS-PAGE) and then transferred onto a polyvinylidene fluoride (PVDF) membrane (Millipore, Bedford, MA, USA). After blocking with 5% non-fat milk, the membrane was incubated overnight at 4 ºC with the primary antibody. The primary antibodies used in our study were as follows: α-SMA, 1:1000; FAP, 1:1000; FSP1, 1;300; vimentin, 1:1000; E-cad, abcam, #ab216833, 1:500; N-cad, abcam, #ab216833, 1:500; FN1, proteintech, 15613-I-AP, 1:500; Slug, Santa Cruz, sc-166476, 1:200; Snail, Santa Cruz, sc-393172, 1:200, and ZEB1, CST, #70512, 1:1000. Then HRP-conjugated secondary antibodies (1:3000) purchased from ZhongShanJinQiao (BeiJing, China) were incubated and the signal was detected with enhanced chemiluminescence.

### RNA extraction and real-time PCR analysis

Total RNA was extracted using TRIzol reagent (Invitrogen, Thermo Fisher Scientific, Waltham, MA, USA) according to the manufacturer's instructions. The purified RNA was reverse-transcribed to form cDNA using a reverse transcriptase kit (TaKaRa, Dalian, China). Quantitative real-time PCR (qRT-PCR) was performed with SYBR Premix ExTaqTM II (TaKaRa) and β-actin as an internal control. The relative expression level of mRNA was normalized to β-actin expression and was calculated using the 2^-ΔΔCt^ method. The primer sequences are shown in Table 1. For exosomal RNA analysis, the exosome concentration was determined by both NTA and BCA assay. 10 µg (2 × 10^8^) exosomes were used for total RNA extraction. 0.5 µg of total RNA was first reverse transcribed with Mir-X™ miRNA First-Strand Synthesis kit and performed qRT-PCR analysis. The U6 small nuclear RNA was used as an internal control.

### MTT assay and colony formation assay

Cell viability was assessed by MTT and colony formation assays as previously described 47. For MTT assays, 20 µL 3-(4, 5-dimethylthiazol-2-yl)-2,5-diphenyltetrazolium bromide (MTT) was added to the medium and the cells were incubated at 37 ºC for 4-6 h. After aspirating the supernatant, 100 mL DMSO was added to each well and absorbance values at 490 nm were measured using a microplate reader (Bio-Rad). For colony formation assays, cell colonies were fixed with methanol and stained with 0.1% crystal violet (Sigma) before the colonies were counted under a microscope.

### Cell migration and Matrigel invasion assays

Cell migration and Matrigel invasion assays were performed as previously described 46. For migration assays, after infiltrating the Transwell for 24-48 h, cells were fixed and stained with 0.1% crystal violet. For invasion assays, the Transwell membrane was coated with Matrigel. The cells on the lower surface were photographed and counted by ImageJ software.

### Lentiviral infection and Matrigel three-dimensional (3D) embedded cultures

Lentiviral packaging was carried out in HEK-293T cells using the 3^rd^-generation system as described previously 48. Lentiviral particles (Lv-mCherry or Lv-EGFP) were harvested at 48 h after transfection. CAFs were labeled with red fluorescent protein by lentivirus infection with Lv-mCherry, while MDA-MB-231 cells were labeled with green fluorescent protein by lentivirus infection with Lv-EGFP. After 3 days, the labeled CAFs and MDA-MB-231 cells were mixed and injected into the semi-solidified Matrigel (BD Biosciences) using a microsyringe. The mixed cell masses were cultured in Matrigel for 7 days and imaged using a fluorescence microscope. The size of the spheroids under a light microscope was calculated according to the following formula: volume = 0.5 × length × width^2^.

### Dual luciferase reporter assay

Cells were co-transfected with pmirGLO plasmids containing the wild-type or mutant USP28 3′-UTR and miRNA mimics using Lipofectamine 2000 (Invitrogen). After 48 h, luciferase activity was determined using the Dual-Luciferase Reporter Assay Kit (Promega, Madison, WI, USA) according to the manufacturer's instructions. Firefly luciferase activity was normalized against *Renilla* luciferase activity.

### *In vivo* tumorigenesis and metastasis assay

Female BALB/c nude mice (aged 4 weeks) were randomly divided into two groups (10 mice per group) and the *in vivo* studies were performed as previously described 49, 50. We established CAFs stably expressing miR-500a-5p by lentivirus infection and selection with geneticin. A total of 3 × 10^6^ MDA-MB-231 cells mixed (1:1) with CAF/miR-500a-5p or CAF/Ctrl cells in 200 μl of PBS: Matrigel at a 3:1 ratio were subcutaneously injected into the flank of nude mice. After the tumors were readily visualized, the tumor growth rate was monitored by measuring tumor diameters every 4 days using a Vernier caliper and the tumor growth curve was recorded accordingly. The tumor volume was calculated according to the following formula: volume = 0.5 × length × width^2^.

A total of 8 × 10^4^ MDA-MB-231 cells stably expressing miR-500a-5p or NC were injected into the lateral tail veins of female BALB/c nude mice (aged 4 weeks) to produce lung metastasis. Six weeks later, lungs were collected and stained with hematoxylin and eosin.

### Immunohistochemistry (IHC)

Tumors from mouse flanks were fixed in formalin for at least 24 h and embedded in paraffin. The paraffin-embedded tissue sections (6 μm thick) were evaluated for the expression of α-SMA and Ki-67 by immunohistochemical (IHC) staining using a streptavidin-peroxidase-biotin reagent kit (Zhongshan Biotechnology, Beijing, China) according to the manufacturer's protocol. Tissue sections were then incubated with streptavidin-HRP complex and then hematoxylin. For the negative control, the antibody solution was replaced with PBS.

### Statistical analyses

Statistical analyses were performed using GraphPad Prism 7.0 (GraphPad Software, San Diego, CA, USA) and PASW Statistics 18.0 (IBM, SPSS, Chicago, IL, USA). Differences between two groups of data and statistical significance were analyzed by Student's *t*-test or one-way analysis of variance (ANOVA). Survival curves were generated using the Kaplan-Meier method, and the differences were assessed by a log-rank test. *P* < 0.05 was considered to indicate a statistical significance, and *P* < 0.01 and *P* < 0.001 were considered to indicate a high degree of statistical significance.

## Supplementary Material

Supplementary figures and tables.

## Figures and Tables

**Table 1:** Primer sequences used for qRT-PCR

Name	Forward sequence	Reverse sequence
USP28	TGAAGCTCTGAAGGCCAGTAA	CTTCCTTGTTGGCAGCACTC
Cdk19	AGCGGGTGGAGGATTTGTTT	TCTCGCAAAAGTGCAATCTCTC
USP13	TCAAAAGATCGCCTGATGAACC	TGATCCAGTTGAAGGCCACC
PTPRD	AACATCCCCCTTCTCCCGAT	GCAAACACTTGATCCACAGGC
DACH1	TCTACACCAAGCTGAAGCGG	TCTAGAACTTGCGTTGGTGC
TMEM170B	ATTACCAGTGCAGCAGTAGCG	ACCTCAAAGTGTAGCGAGGA
FOXP1	CGGGCGTGAAGGCGG	ACAGAGGGAAGCCTTTTGGC
TXNRD1	CCGCCGTAGGTCAGCTAAAG	CGTGTGCATGTGGACCTACT
CDH1	GCTGGACCGAGAGAGTTTCC	CAAAATCCAAGCCCGTGGTG
ATG12	GGGAAGGACTTACGGATGTCTC	AGGAGTGTCTCCCACAGCCTTT
NKX3-1	CGCAGAACGACCAGCTGAGCA	CCTGAAGTGTTTTCAGAGTCCAAC
CCNC	GCAGAAAGATGCCAGGCAATGG	CTCTCATCGAAATTCTTCCACTGC
BLL10	CACCCTTGTTGAATCTATTCGGC	GAGGTTGTTCGTGGCTCCATCT
TBL1XR1	GAGAACAGCACCAGTGGCTCTA	CCATCATAGGAACCAGTTGCTAG

## Abbreviations

CAFs: cancer-associated fibroblasts
USP28: Ubiquitin specific peptidase 28
EMT: epithelial-to-mesenchymal transition
NFs: normal fibroblasts
CM: conditioned media
α-SMA: α-smooth muscle actin
FAP: fibroblast activation protein
FSP1: fibroblast specific protein 1
GFP: Green fluorescent protein
TCGA: The Cancer Genome Atlas
ISH: *in situ* hybridization
NC: negative control
WT: wild-type
siRNAs: small interfering RNAs

## References

[1] Bray F, Ferlay J, Soerjomataram I, Siegel RL, Torre LA, Jemal A (2018). Global cancer statistics 2018: GLOBOCAN estimates of incidence and mortality worldwide for 36 cancers in 185 countries. CA Cancer J Clin.

[2] Wang M, Zhao J, Zhang L, Wei F, Lian Y, Wu Y (2017). Role of tumor microenvironment in tumorigenesis. J Cancer.

[3] Quail DF, Joyce JA (2013). Microenvironmental regulation of tumor progression and metastasis. Nat Med.

[4] Joyce JA, Pollard JW (2009). Microenvironmental regulation of metastasis. Nat Rev Cancer.

[5] Mao Y, Keller ET, Garfield DH, Shen K, Wang J (2013). Stromal cells in tumor microenvironment and breast cancer. Cancer Metastasis Rev.

[6] Richards KE, Zeleniak AE, Fishel ML, Wu J, Littlepage LE, Hill R (2017). Cancer-associated fibroblast exosomes regulate survival and proliferation of pancreatic cancer cells. Oncogene.

[7] Gandellini P, Andriani F, Merlino G, D'Aiuto F, Roz L, Callari M (2015). Complexity in the tumour microenvironment: Cancer associated fibroblast gene expression patterns identify both common and unique features of tumour-stroma crosstalk across cancer types. Semin Cancer Biol.

[8] Sun Q, Zhang B, Hu Q, Qin Y, Xu W, Liu W (2018). The impact of cancer-associated fibroblasts on major hallmarks of pancreatic cancer. Theranostics.

[9] Lobb RJ, Lima LG, Moller A (2017). Exosomes: Key mediators of metastasis and pre-metastatic niche formation. Semin Cell Dev Biol.

[10] Mathivanan S, Ji H, Simpson RJ (2010). Exosomes: extracellular organelles important in intercellular communication. J Proteomics.

[11] Mashouri L, Yousefi H, Aref AR, Ahadi AM, Molaei F, Alahari SK (2019). Exosomes: composition, biogenesis, and mechanisms in cancer metastasis and drug resistance. Mol Cancer.

[12] Qin X, Guo H, Wang X, Zhu X, Yan M, Wang X (2019). Exosomal miR-196a derived from cancer-associated fibroblasts confers cisplatin resistance in head and neck cancer through targeting CDKN1B and ING5. Genome Biol.

[13] Au Yeung CL, Co NN, Tsuruga T, Yeung TL, Kwan SY, Leung CS (2016). Exosomal transfer of stroma-derived miR21 confers paclitaxel resistance in ovarian cancer cells through targeting APAF1. Nat Commun.

[14] Sun Z, Shi K, Yang S, Liu J, Zhou Q, Wang G (2018). Effect of exosomal miRNA on cancer biology and clinical applications. Mol Cancer.

[15] Wu T, Dai Y (2017). Tumor microenvironment and therapeutic response. Cancer Lett.

[16] Mueller MM, Fusenig NE (2004). Friends or foes - bipolar effects of the tumour stroma in cancer. Nat Rev Cancer.

[17] Costea DE, Hills A, Osman AH, Thurlow J, Kalna G, Huang X (2013). Identification of two distinct carcinoma-associated fibroblast subtypes with differential tumor-promoting abilities in oral squamous cell carcinoma. Cancer Res.

[18] Fiori ME, Di Franco S, Villanova L, Bianca P, Stassi G, De Maria R (2019). Cancer-associated fibroblasts as abettors of tumor progression at the crossroads of EMT and therapy resistance. Mol Cancer.

[19] Bhowmick NA, Neilson EG, Moses HL (2004). Stromal fibroblasts in cancer initiation and progression. Nature.

[20] Karakasheva TA, Lin EW, Tang Q, Qiao E, Waldron TJ, Soni M (2018). IL-6 Mediates Cross-Talk between Tumor Cells and Activated Fibroblasts in the Tumor Microenvironment. Cancer Res.

[21] Wen S, Hou Y, Fu L, Xi L, Yang D, Zhao M (2019). Cancer-associated fibroblast (CAF)-derived IL32 promotes breast cancer cell invasion and metastasis via integrin beta3-p38 MAPK signalling. Cancer Lett.

[22] Qin X, Yan M, Wang X, Xu Q, Wang X, Zhu X (2018). Cancer-associated Fibroblast-derived IL-6 Promotes Head and Neck Cancer Progression via the Osteopontin-NF-kappa B Signaling Pathway. Theranostics.

[23] Yang X, Li Y, Zou L, Zhu Z (2019). Role of Exosomes in Crosstalk Between Cancer-Associated Fibroblasts and Cancer Cells. Front Oncol.

[24] Halvorsen AR, Helland A, Gromov P, Wielenga VT, Talman MM, Brunner N (2017). Profiling of microRNAs in tumor interstitial fluid of breast tumors - a novel resource to identify biomarkers for prognostic classification and detection of cancer. Mol Oncol.

[25] Lima CR, Gomes CC, Santos MF (2017). Role of microRNAs in endocrine cancer metastasis. Mol Cell Endocrinol.

[26] O'Bryan S, Dong S, Mathis JM, Alahari SK (2017). The roles of oncogenic miRNAs and their therapeutic importance in breast cancer. Eur J Cancer.

[27] Yang F, Ning Z, Ma L, Liu W, Shao C, Shu Y (2017). Exosomal miRNAs and miRNA dysregulation in cancer-associated fibroblasts. Mol Cancer.

[28] Wang JW, Wu XF, Gu XJ, Jiang XH (2019). Exosomal miR-1228 From Cancer-Associated Fibroblasts Promotes Cell Migration and Invasion of Osteosarcoma by Directly Targeting SCAI. Oncol Res.

[29] Bao L, Zhang M, Han S, Zhan Y, Guo W, Teng F (2018). MicroRNA-500a Promotes the Progression of Hepatocellular Carcinoma by Post-Transcriptionally Targeting BID. Cell Physiol Biochem.

[30] Guo Y, Chen L, Sun C, Yu C (2017). MicroRNA-500a promotes migration and invasion in hepatocellular carcinoma by activating the Wnt/beta-catenin signaling pathway. Biomed Pharmacother.

[31] Zhao Y, Wang Y, Wang Y (2017). Up-regulated miR-500a enhances hepatocarcinoma metastasis by repressing PTEN expression. Biosci Rep.

[32] Zhang L, Ding Y, Yuan Z, Liu J, Sun J, Lei F (2015). MicroRNA-500 sustains nuclear factor-kappaB activation and induces gastric cancer cell proliferation and resistance to apoptosis. Oncotarget.

[33] Tang W, Zhou W (2019). The p300/YY1/miR-500a-5p/HDAC2 signalling axis regulates cell proliferation in human colorectal cancer. Nat Commun.

[34] Degli Esposti D, Aushev VN (2017). miR-500a-5p regulates oxidative stress response genes in breast cancer and predicts cancer survival. Sci Rep.

[35] Esquela-Kerscher A, Slack FJ (2006). Oncomirs - microRNAs with a role in cancer. Nat Rev Cancer.

[36] Wang X, Liu Z, Zhang L, Yang Z, Chen X, Luo J (2018). Targeting deubiquitinase USP28 for cancer therapy. Cell Death Dis.

[37] Popov N, Wanzel M, Madiredjo M, Zhang D, Beijersbergen R, Bernards R (2007). The ubiquitin-specific protease USP28 is required for MYC stability. Nat Cell Biol.

[38] Diefenbacher ME, Popov N, Blake SM, Schulein-Volk C, Nye E, Spencer-Dene B (2014). The deubiquitinase USP28 controls intestinal homeostasis and promotes colorectal cancer. J Clin Invest.

[39] Popov N, Herold S, Llamazares M, Schulein C, Eilers M (2007). Fbw7 and Usp28 regulate myc protein stability in response to DNA damage. Cell Cycle.

[40] Diefenbacher ME, Chakraborty A, Blake SM, Mitter R, Popov N, Eilers M (2015). Usp28 counteracts Fbw7 in intestinal homeostasis and cancer. Cancer Res.

[41] Schulein-Volk C, Wolf E, Zhu J, Xu W, Taranets L, Hellmann A (2014). Dual regulation of Fbw7 function and oncogenic transformation by Usp28. Cell Rep.

[42] Cao C, Vasilatos SN, Bhargava R, Fine JL, Oesterreich S, Davidson NE (2017). Functional interaction of histone deacetylase 5 (HDAC5) and lysine-specific demethylase 1 (LSD1) promotes breast cancer progression. Oncogene.

[43] Richter K, Paakkola T, Mennerich D, Kubaichuk K, Konzack A, Ali-Kippari H (2018). USP28 Deficiency Promotes Breast and Liver Carcinogenesis as well as Tumor Angiogenesis in a HIF-independent Manner. Mol Cancer Res.

[44] Ying W, Riopel M, Bandyopadhyay G, Dong Y, Birmingham A, Seo JB (2017). Adipose Tissue Macrophage-Derived Exosomal miRNAs Can Modulate *In vivo* and *In vitro* Insulin Sensitivity. Cell.

[45] Wang X, Zhang H, Yang H, Bai M, Ning T, Deng T (2020). Exosome-delivered circRNA promotes glycolysis to induce chemoresistance through the miR-122-PKM2 axis in colorectal cancer. Mol Oncol.

[46] Liang Y, Song X, Li Y, Su P, Han D, Ma T (2019). circKDM4C suppresses tumor progression and attenuates doxorubicin resistance by regulating miR-548p/PBLD axis in breast cancer. Oncogene.

[47] Sang Y, Chen B, Song X, Li Y, Liang Y, Han D (2019). circRNA_0025202 Regulates Tamoxifen Sensitivity and Tumor Progression via Regulating the miR-182-5p/FOXO3a Axis in Breast Cancer. Mol Ther.

[48] Chen B, Zhao L, Li X, Ji YS, Li N, Xu XF (2014). Syntaxin 8 modulates the post-synthetic trafficking of the TrkA receptor and inflammatory pain transmission. J Biol Chem.

[49] Zhang Z, Li X, Sun W, Yue S, Yang J, Li J (2017). Loss of exosomal miR-320a from cancer-associated fibroblasts contributes to HCC proliferation and metastasis. Cancer Lett.

[50] Tang X, Hou Y, Yang G, Wang X, Tang S, Du YE (2016). Stromal miR-200s contribute to breast cancer cell invasion through CAF activation and ECM remodeling. Cell Death Diff.

