# Supplementary material for: Exosomal miR-500a-5p derived from cancer-associated fibroblasts promotes breast cancer cell proliferation and metastasis through targeting USP28
Source: Theranostics. 2021 Feb 6;11(8):3932–47. doi: 10.7150/thno.53412 (PMC7914354; doi:10.7150/thno.53412)
Supplement: Supplementary file 1 — Supplementary figures and tables. [file thnov11p3932s1.pdf]

Supplementary Figure Legend:

Supplementary Figure 1. CAF-mediated promotion of breast cancer cell proliferation and migration is in an exosome-dependent manner. After the breast cancer cells was treated with the EV-free conditioned medium of NFs or CAFs after ultracentrifugation, MTT **(A)** and transwell **(B)** assay were performed. Data are presented as means ± SEM (Student's t-test).

Supplementary Figure 2. Scratch wound healing assays. The assays were performed in MDA-MB-231 cells **(A)** and MCF7 cells **(B)**. **(C)** Quantification of the wound area in (A) and (B). Data are presented as means ± SEM ($^{\#\#}P < 0.01$; $^{***}P < 0.001$; Student's t-test).

Supplementary Figure 3. USP28 inhibition increases breast cancer cell proliferation and metastasis. (A) The interference efficiency of USP28 siRNAs was detected by Western blot assay. MTT **(B)** and transwell **(C)** assays were performed in MDA-MB-231 and MCF7 cells in which USP28 was inhibited. Data are presented as means ± SEM ($^{*}P < 0.05$; $^{**}P < 0.01$; $^{***}P < 0.001$; Student's t-test).

Supplementary Figure 4. Quantification of western blotting in Figure 5 (E) and Figure 6 (L). **(A)** Quantification of western blotting in Figure 5 (E). **(B)** Quantification of western blotting in Figure 6 (L). Data are presented as means ± SEM ($^{\#,*}P < 0.05$; $^{\#\#,**}P < 0.01$; $^{***}P < 0.001$; Student's t-test).

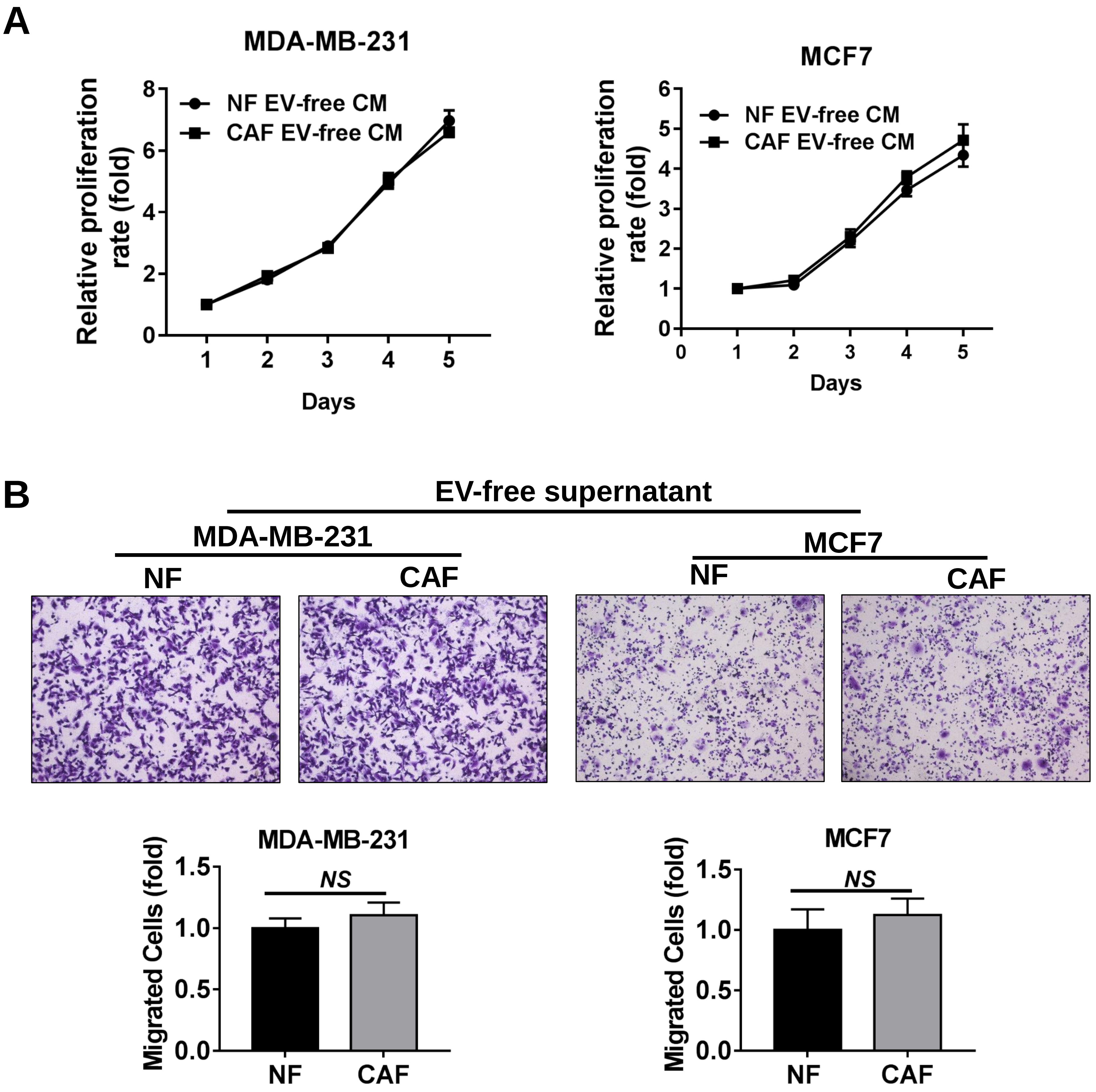

Supplementary Figure 1

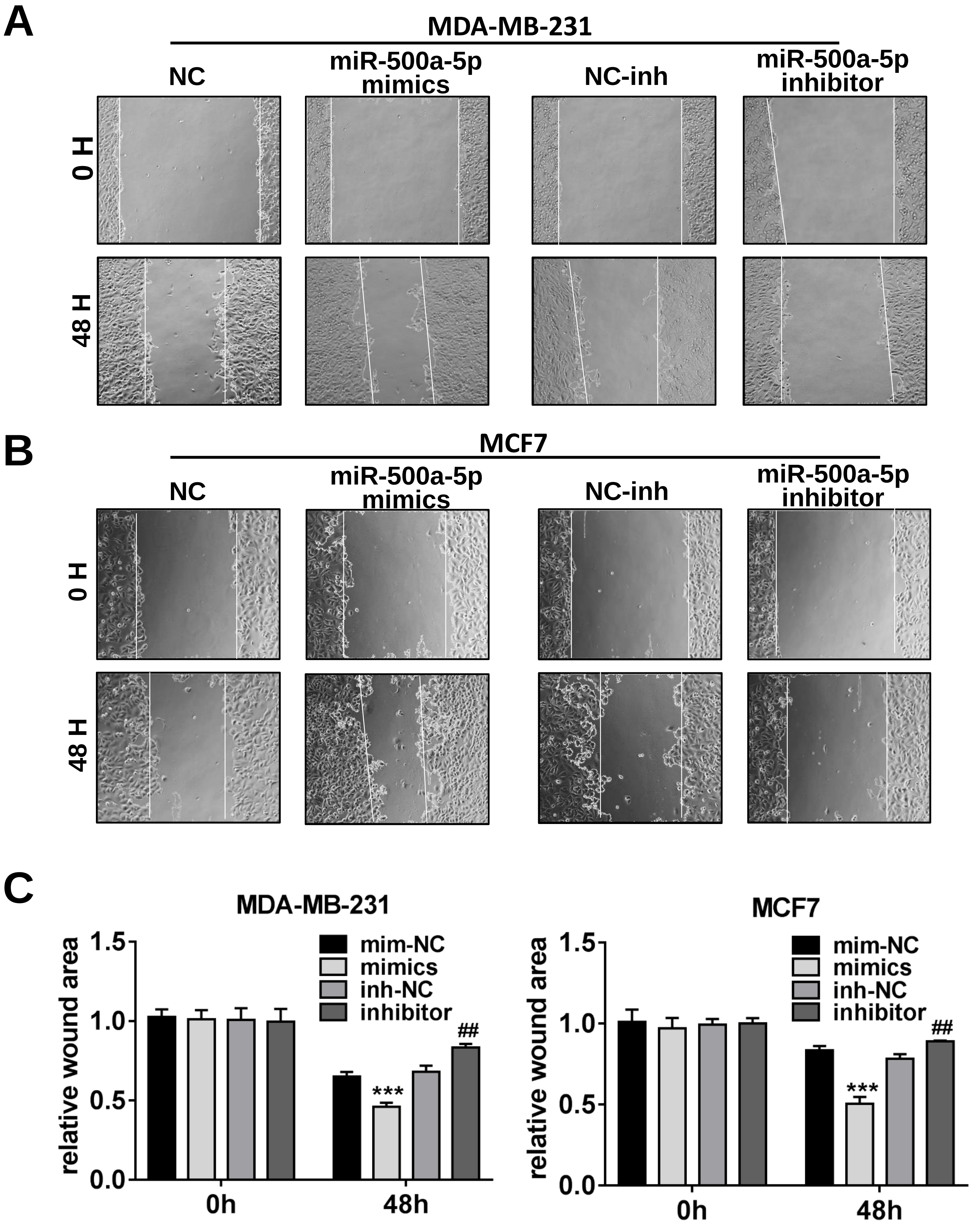

Supplementary Figure 2

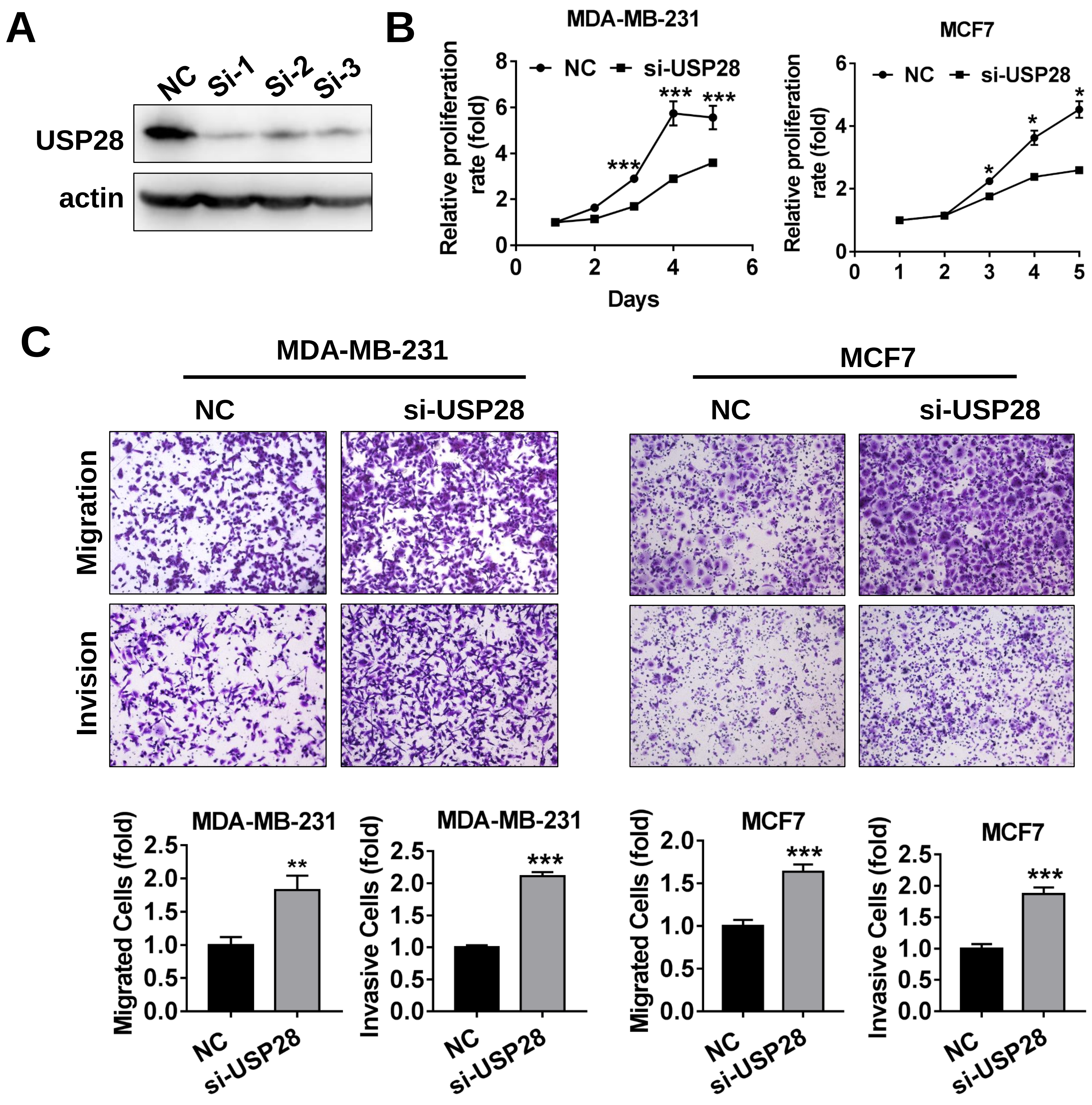

Supplementary Figure 3

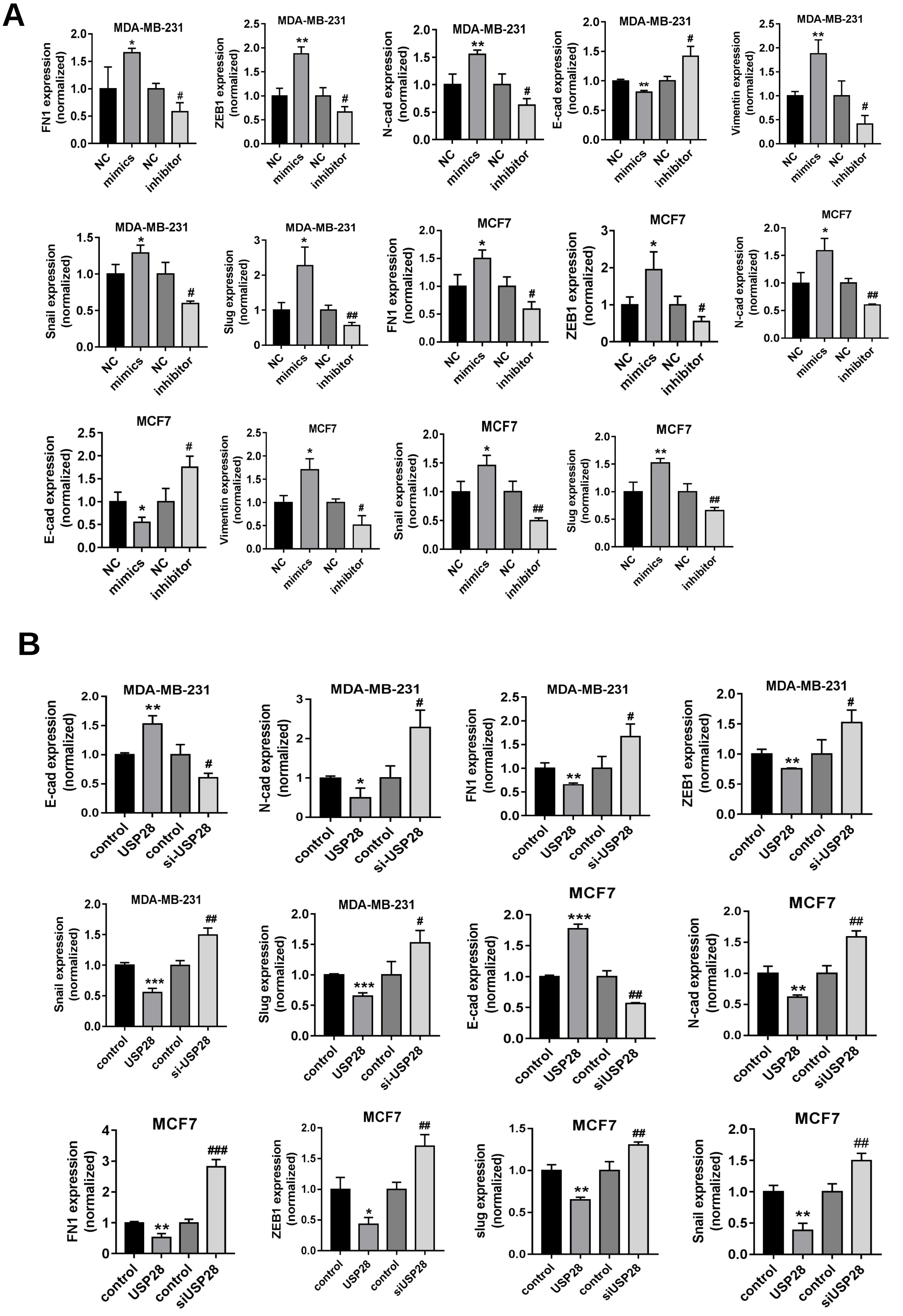

Supplementary Figure 4
